# Effects of Diet Supplemented With Hydrolyzable Tannin on the Growth Performance, Antioxidant Capacity, and Muscle Nutritional Quality of Juvenile *Mastacembelus armatus*

**DOI:** 10.1155/2024/8266189

**Published:** 2024-11-04

**Authors:** Xiaowen Xue, Yiman Chen, Zhide Yu, Yuwei Feng, Linan Zhang, Chong Han, Xiaoli Yin, Baoyue Lu, Hu Shu

**Affiliations:** School of Life Sciences, Guangzhou University, Guangzhou 510006, China

**Keywords:** growth, hepatopancreatic health, hydrolyzable tannin, *Mastacembelus armatus*, muscle quality

## Abstract

In this study, four groups of diet were prepared, with eel commercial diet without hydrolyzable tannin (HT) as the control group (H0), and the other three groups were fed with diet containing 0.05% (H1), 0.1% (H2), and 0.2% (H3) doses of HT to juvenile *Mastacembelus armatus* with an initial body weight of (0.40 ± 0.005) g. Juvenile fish in all groups were fed continuously for 60 days. Growth indices, hepatopancreatic antioxidant enzymes, biochemical indices (including total superoxide dismutase [T-SOD], catalase [CAT], malondialdehyde [MDA], total antioxidant capacity [T-AOC], alanine aminotransferase [ALT], aspartate aminotransferase [AST], alkaline phosphatase [AKP], and triglyceride [TG]), the content of muscle amino acids and fatty acids, stomach and intestine enzyme activities (pepsin, amylase, lipase), and genes expressions were evaluated. The results showed that 0.1% HT significantly improved the growth performance, hepatopancreatic antioxidant capacity, as well as muscle quality and lipase activity of juvenile *M. armatus*. In summary, the optimal addition level of HT in the diet of juvenile *M. armatus* is 0.1%, which helps to improve aquaculture efficiency and improve the muscle quality of *M. armatus*. However, the long-term effects of feeding HT on *M. armatus* and its physiological reaction mechanism need to be further explored.

## 1. Introduction

*Mastacembelus armatus*, belongs to Symbranchiformes, Mastacembelidae, and *Mastacembelus*, mainly distributed in Southeast Asia and Southern China. Due to its delicious meat, rich content of various amino acids, and unsaturated fatty acids in muscles, it is highly favored by consumers [1, 2]. In recent years, due to overfishing and environmental pollution, the wild resources of *M. armatus* have been seriously damaged. Fujian, Yunnan, and Guangdong Provinces in China have listed *M. armatus* as one of the key protected wild aquatic animals [1]. Therefore, it is necessary to artificially cultivate and breeding the *M. armatus*. However, the frequent occurrence of diseases has become an important factor restricting the healthy development of the *M. armatus* aquaculture industry. Nevertheless, the frequent use of antibiotics in aquaculture often leads to the development of drug resistance, which is incompatible with the requirements of sustainable and healthy aquaculture. The use of antibiotics is being strictly controlled in many countries [3]. Therefore, the search for environmentally friendly antibiotic alternatives that cannot only replace antibiotics with energy efficiency, prevent disease occurrence, but also promote animal growth has become one of the hot topics in animal nutrition research [4, 5].

Tannins, a polyphenolic natural product widely found in plants, are often used as antinutritional factors because of their astringency, poor taste, and negative impact on nutrient utilization [6]. However, following in-depth research on tannins, it has been found that the effectiveness of tannins on animals depends on the quantity and type of tannins added [7]. Low concentrations of tannins usually have positive effects on animals. For example, 0.15% hydrolyzable tannins (HTs) could significantly improve the growth performance, antioxidant capacity, intestinal microflora, and resistance against *Vibrio parahaemolyticus* of *Litopenaeus vannamei* [8]. In total, 0.062% condensed tannins (CTs) increased plasma antioxidant enzyme activity and *Nrf2* gene expression as well as intestinal immune function of *Micropterus salmoides* [9]. In contrast, high concentrations of tannins may have negative effects on animals. For example, 0.31% CTs inhibited the organism's antioxidant capacity and immune function of *M. salmoides* [9]. Adding more than 1% tannic acid to the diet of juvenile *Dicentrarchus labrax* L. could weaken its growth performance [10]. Based on the differences in molecular structure and chemical properties, tannins can be classified into two types: HTs and CTs [11]. HTs can be composed of ellagic acid and its derivatives containing sugar groups or gallic acid esters, with a relative molecular mass of 500–3000 Da. The molecule contains neighboring phenolic hydroxyl groups, which are easily oxidized, and is an excellent hydrogen donor, able to scavenge free radicals effectively [12–14]. HTs can be classified into two main categories, ellagitannins and gallotannins, based on the differences in the phenolic carboxylic acids produced upon hydrolysis. Ellagitannins release hexahydroxydiphenic acid during hydrolysis, which is eventually converted into ellagic acid, while gallotannins (also known as pentagallate tannins) produce gallic acid after hydrolysis [15]. HTs are natural, safe, and versatile feed additives with significant growth-promoting effects, as well as antioxidant, bacteriostatic, anti-inflammatory, and antiviral bioactivities [8, 12]. These characteristics make HTs as high-quality diet additives and promising alternatives to antibiotics.

At present, there is no special formula diet for the *M. armatus*, and the widely available eel commercial diet is generally used as the formula diet for *M. armatus* in actual production. No diet additive formulations have been developed to promote the growth and health of *M. armatus*. In this study, the effects of HT on the growth performance, hepatopancreatic health, muscle quality, and digestive enzyme activities of juvenile *M. armatus* were investigated to determine the optimal amount of HT in the diet of *M. armatus* and to provide ideas for solving the breeding problem of *M. armatus* with slow growth and weak disease resistance. In addition, this study would provide a robust scientific foundation for the utilization of HTs in enhancing fish growth and resilience to disease, reduce the reliance on traditional growth-promoting antibiotics, reduce environmental contamination, and promote the sustainable and healthy development of the aquaculture industry.

## 2. Materials and Methods

### 2.1. Experimental Diet Preparation

The basic diet used in this study was eel commercial diet (powdered diet), purchased from Dongguan Yinhua Biotechnology Co., Ltd. Its nutritional level is shown in Table 1. The HT was purchased from Guangzhou Anning Biotechnology Co. The groups were defined according to the amount of HT, with the control group designated as H0 (basal diet), the experimental groups as H1 (basal diet + 0.05% HT), H2 (basal diet + 0.1% HT), and H3 (basal diet + 0.2% HT), respectively. The diet preparation method for the test groups was as follows: 12.5 g of HT powder was dissolved in 100 mL of anhydrous ethanol to form 125 mg/mL of HT mother liquor. This was then diluted in anhydrous ethanol and evenly sprayed on the basic diet, resulting in final diet concentrations of HT of 0.05%, 0.1%, and 0.2%, respectively. The diet was stirred evenly. The mixed diet was placed in the oven at 50°C for drying and subsequently transferred to the refrigerator at 4°C for storage. Before feeding the juvenile *M. armatus*, add appropriate amount of water (diet:water = 1 : 0.7) and knead the diet into a dough shape. Diet dough was not easily dissolved in water and would not cause large losses.

### 2.2. Experimental Animal Feeding Management

The aquaculture experiments were conducted at Guangdong Lianyi Aquatic Technology Co., Ltd., in Jiangmen City, Guangdong Province, China, and the juvenile *M. armatus* were purchased from Guangdong Tenghai Aquaculture Co., Ltd. The experimental fish were transported to the aquaculture base and placed in sterilized holding tanks for 1 week, during which they were fed with eel commercial diet. After normal feeding, the juvenile *M. armatus* with the similar size, good health condition, and initial weight of (0.40 ± 0.005) g were selected and randomly divided into 12 tanks (four groups, three replicates in each group), with 33 fish in each tank. Apparent satiation feeding was used, feeding twice a day in the morning and evening (8 : 00 and 18 : 00), siphoning method was used to remove feces after 2 h of feeding, and half of the water was changed once every 3 days, and the water source used was the well water that had been aerated for 48 h. During the experiment, the aquaculture water was continuously aerated, and the water temperature was maintained at 28 ± 2°C with the pondus hydrogenii (pH) value of 6.8–7.8. The culture time was 60 days, and daily feeding and mortality rates were recorded. Animal experiments were conducted in accordance with the regulations of the Guide for Care and Use of Laboratory Animals and were approved by the Committee of Laboratory Animal Experimentation at Guangzhou University.

### 2.3. Sample Collection and Analysis

After the experiment, all experimental fish were fasted for 24 h, 10 *M. armatus* were randomly selected from each culture tank and anesthetized with eugenol (100 ppm) [9]. Their weights and lengths were measured and recorded. The intact viscera masses were taken and weighed after draining the surface water. The brain, back muscles, intact hepatopancreas, stomach, and intestines were separated. All tissues were placed in sterile, enzyme-free centrifuge tubes, and stored briefly in liquid nitrogen. Subsequently, all samples were stored at −80°C for subsequent assay analysis.

#### 2.3.1. Measurement of Growth Performance

The formulae for growth performance-related indicators are as follows:

  $$\begin{array}{c} \text{Survival rate }\left(\text{SR},\%\right)=\text{Number of survival fish}/\text{initial number of fish}\times 100, \end{array}$$

  $$\begin{array}{c} \text{Weight gain rate }\left(\text{WGR},\%\right)=\left(\text{Final body weight }\left(g\right)-\text{initial body weight }\left(g\right)\right)/\text{initial body weight }\left(g\right)\times 100, \end{array}$$

  $$\begin{array}{c} \text{Specific growth rate }\left(\text{SGR},\%/\text{day}\right)=\left(\left(\text{Ln final body weight}-\text{ln initial body weight}\right)/\text{number of days in the growth period}\right)\times 100, \end{array}$$

  $$\begin{array}{c} \text{Feed coefficient ratio }\left(\text{FCR}\right)=\text{Total feed supplied }\left(g\right)/\left(\text{final body weight}-\text{initial body weight}\right)\left(g\right), \end{array}$$

  $$\begin{array}{c} \text{Condition factor }\left(\text{CF},g/{\text{cm}}^{3}\right)=\left(\text{Body weight }\left(g\right)/\text{body length }\left({\text{cm}}^{3}\right)\right)\times 100, \end{array}$$

  $$\begin{array}{c} \text{Visceral somatic index }\left(\text{VSI},\%\right)=\text{Visceral weight }\left(g\right)/\text{body weight }\left(g\right). \end{array}$$

#### 2.3.2. Determination of Hepatopancreatic Antioxidant and Biochemical Indices

The samples of hepatopancreatic tissues were weighed and homogenized by adding 0.9% saline at a ratio of weight (g) to body weight (mL) of 1 : 9. The homogenates were then centrifuged at 2500 r/m for 10 min, after which the supernatant was taken and diluted. The optical density (OD) values were determined using an ultraviolet–visible spectrophotometer. The following biochemical parameters were determined using the appropriate kits: total superoxide dismutase (T-SOD; detection wavelength, 550 nm), catalase (CAT; detection wavelength, 405 nm), malondialdehyde (MDA; detection wavelength, 532 nm), total antioxidant capacity (T-AOC; detection wavelength, 520 nm), alanine aminotransferase (ALT; detection wavelength, 505 nm), aspartate aminotransferase (AST; detection wavelength, 505 nm), triglyceride (TG; detection wavelength, 500 nm), and alkaline phosphatase (AKP; detection wavelength, 520 nm). The kits were provided by Nanjing Jiancheng Biological Engineering Co.

#### 2.3.3. Quantitative Real-Time Polymerase Chain Reaction (PCR)

The brains, hepatopancreas, and muscle samples of H0, H1, H2, and H3 stored at −80°C were subjected to total ribonucleic acid (RNA) extraction using the RNA isolater total RNA extraction reagent (Vazyme, China). The integrity, concentration, and purity of the RNA were assessed through electrophoresis and microspectrophotometer analysis. An OD260/OD280 ratio of ~1.8–2.0 was considered indicative of usable RNA. The HiScript II Q RT SuperMix for qPCR (+gDNA [genomic deoxyribonucleic acid] wiper) (Vazyme, China) was utilized for reverse transcription of the target tissues' RNA into complementary deoxyribonucleic acid (cDNA) in juvenile *M. armatus*. Quantitative real-time PCR was performed on the Roche 480 real-time PCR system according to the ChamQ SYBR qPCR Master Mix kit (Vazyme, China) instructions. Primers for*β-actin* (ncbi_113140763), insulin-like growth factor (IGF) 1 (*igf-1*, ncbi_113141817), IGF 2 (*igf-2*, ncbi_113132952), growth hormone (GH) (*gh*, ncbi_113140986), growth hormone-releasing hormone (GHRH) (*ghrh*, ncbi_113130746), CAT (*cat*, ncbi_113133383), cu/zn-superoxide dismutase (*cu/zn-sod*, ncbi_113143408), glutathione S-transferases *α* (*gstα*, ncbi_113131556), myogenic differentiation (*myod*, ncbi_113132922), myogenin (*myog*, ncbi_113129866), myogenic factor 5 (*myf5*, ncbi_113142072), myogenic factor 6 (*myf6*, ncbi_113142052), and myostatin (*mstn*, ncbi_113123823) were designed using National Center for Biotechnology Information (NCBI) Prime-BLAST Primer sequence (Table 2). These primers were synthesized by Guangzhou Shengong Biotechnology Co. *β-Actin* served as the internal reference gene with CT values normalized across all samples. Gene expression levels were calculated using the 2^−*ΔΔ*CT^ method with H0 group mRNA as a baseline [16], and GraphPad Prism 8 was used to visualize the experimental results.

#### 2.3.4. Muscle Amino Acids

Three replicate samples were taken in each group, for a total of 12 samples in four groups. The amino acid content in the sample was determined using a 1290 Infinity II series UHPLC System (Agilent, USA) and a 6460 Triple Quadrupole Mass Spectrometer (Agilent, USA). The procedure was as follows: 20 mg of sample was weighed, 1000 µL of extraction solution (V_acetonitrile_ : V_methanol_ : V_water_ = 2 : 2 : 1) was added and mixed, then ground for 4 min and sonicated in an ice–water bath for 5 min. The samples were allowed to stand at −40°C for 1 h. The samples were centrifuged at 4°C (12,000 rpm for 15 min) and the supernatants were removed for UHPLC-MS/MS analysis [17].

#### 2.3.5. Muscle Fatty Acids

Three replicate samples were taken in each group, for a total of 12 samples in four groups. The fatty acid content of the samples was determined using a 7890 B gas chromatograph (Agilent, USA) and a 5977 B mass spectrometer (Agilent, USA). The procedure was as follows: 25 mg of the sample was weighed, 500 µL of extraction solution (V_isopropanol_ : V_hexane_ = 2 : 3) was added and mixed, then ground for 4 min and sonicated in an ice–water bath for 5 min. The supernatant was removed by centrifugation at 4°C (12,000 rpm, 15 min). To the remaining mixture, 500 µL of extract was added, mixed well, placed in an ice–water bath, and centrifuged to remove the supernatant as described above. The supernatant (800 µL) obtained by mixing twice was dried with nitrogen. Add 500 µL of the mixture (V_methanol_ : V_trimethylsilyl diazomethane_ = 1 : 2), leave for 30 min and dry again with nitrogen. Added hexane (160 µL) and the supernatant was centrifuged (12,000 rpm, 1 min) for gas chromatography–mass spectrometry (GC–MS) [18].

#### 2.3.6. Digestive Enzyme Activity Analysis

The tissue and 0.9% sterile saline solution (w/v = 1 : 9) were frozen homogenized centrifuged at 2500 rpm for 10 min to remove the supernatant, and the assay was completed within 12 h. The activities of pepsin (detection wavelength, 660 nm), amylase (detection wavelength, 660 nm), and lipase (detection wavelength, 420 nm) were tested using the reagent kit from Nanjing Jiancheng Biological Engineering Co., according to the manufacturer's instructions [19, 20].

### 2.4. Data Processing and Analysis

The experimental data were expressed in the form of mean ± standard error (SE). Significant differences in the data were tested by one-way analysis of variance (ANOVA) followed by Duncan's multiple comparisons test. *p* < 0.05 was considered as statistically significant. All analyses were performed using International Business Machines Corporation Statistical Product and Service Solutions (IBM SPSS) Statistics 26.0 software. In the following results, peer data with different superscripts indicated a significant difference, while no superscript or the same superscript indicated no significant difference.

## 3. Results

### 3.1. Growth Performance Analysis

The results showed that after 60 days of culture, the growth performance of juvenile *M. armatus* in the H1 and H2 groups was significantly improved compared to the H0 group. Final body weight (FBW), WGR, SGR, and SR were significantly increased, FCR was significantly decreased (*p*  < 0.05), and the growth advantage of H2 group was more obvious than that of H1 group. SR was significantly higher and FCR was significantly lower (*p*  < 0.05) in group H3 compared with group H0, but there were no significant differences (*p*  > 0.05) in FBW, WGR, SGR, and CF (Table 3). Therefore, feeding 0.1% HT (H2) could significantly improve the growth of juvenile *M. armatus*.

### 3.2. Hepatopancreatic Antioxidant Capacity and Biochemical Indices

The effects of HT on hepatopancreatic antioxidant capacity and biochemical indices of juvenile *M. armatus* are shown in Table 4. T-SOD activity was significantly higher in both H2 and H3 groups than that in H0 and H1 groups (*p*  < 0.05), whereas there was no significant difference between H0 and H1 groups (*p*  > 0.05). CAT activity was significantly lower, while MDA was significantly higher in the H0 group than that in the other three groups (*p*  < 0.05). T-AOC activity was significantly higher in H1 and H2 groups than that in H0 and H3 groups (*p*  < 0.05), and there was no significant difference between H1 and H2 groups and between H0 and H3 groups (*p*  > 0.05). ALT activity was significantly higher in H2 and H3 groups than that in H0 and H1 groups (*p*  < 0.05), with no significant differences between H2 and H3 groups and between H0 and H1 groups (*p*  > 0.05). With increasing HT addition (0%, 0.05%, 0.1%, and 0.2%), the AST showed a tendency to increase and then decrease, with the highest activity in the H2 group, which was significantly higher than that of H0, H1, and H3 groups (*p*  < 0.05), and there was no significant difference between the latter three groups (*p*  > 0.05). The activity of AKP in H1, H2, and H3 groups was significantly higher than that in H0 group, and the activity was strongest in H2 group (*p*  < 0.05). TG content was significantly lower in H0 group than that in the HT-added groups (*p*  < 0.05), and there was no significant difference between H1, H2, and H3 groups (*p*  > 0.05). Therefore, HT can promote the healthy growth of *M. armatus* by enhancing the antioxidant capacity and lipid anabolism of hepatopancreas.

### 3.3. Muscle Quality Identification

#### 3.3.1. Amino Acid Composition Analysis

The amino acid composition in the muscle of *M. armatus* is shown in Table 5, with a total of 17 amino acids detected (Table 5). Dietary HT had no significant effect on the content of total essential amino acids (EAAs) and total nonessential amino acids (NEAAs) in the muscle of juvenile *M. armatus*. However, HT groups exhibited a significantly higher content of valine (Val) than the control group (*p*  < 0.05). The content of methionine (Met) was significantly higher in the H3 group than that in the H0 group (*p*  < 0.05). Furthermore, the content of Met and histidine (His) in H1 and H2 groups was higher than that in H0 group, although the difference was not statistically significant (*p*  > 0.05). The glycine (Gly) content of the HT groups was found to be higher than that of the control group (H0), although this difference was not statistically significant (*p*  > 0.05). No significant difference in Glu content between the HT groups and the control group, but the H3 group exhibited a higher content than the H0 group (*p*  > 0.05). Alanine (Ala) content in H1 and H2 groups was higher than that in H0 group, but there was no significant difference (*p*  > 0.05). The H3 group exhibited a significantly higher level of Ala than the H0 group (*p*  < 0.05). Tyrosine (Tyr) content in the H3 group was also significantly higher than that in the H0 group (*p*  < 0.05). These results indicate that although feeding HT to *M. armatus* has no significant effect on the total EAAs and NEAAs content in muscle, 0.1% HT (H2) can increase the content of EAAs Val, Met, and His, as well as NEAAs, Ala, and Gly.

#### 3.3.2. Fatty Acids Composition

A total of 28 fatty acids were identified in the muscle of juvenile *M. armatus* (Table 6). The total amount of saturated fatty acids (ΣSFAs) was found to be significantly higher in the H2 group than that in the H0 group (*p*  < 0.05). However, there was no significant difference in ΣSFA content among H0, H1, and H3 groups (*p*  > 0.05). The total amount of monounsaturated fatty acids (ΣMUFAs) was significantly higher in the H2 group compared to the H0 group (*p*  < 0.05). The content of oleic acid (C18 : 1n9) was significantly higher than that in the H0 group (*p*  < 0.05). The total amount of polyunsaturated fatty acids (ΣPUFAs), *n*−3 series PUFAs (Σ*n*−3 PUFAs) and *n*−6 series PUFAs (Σ*n*−6 PUFAs) were significantly higher in the H2 group than that in the H0 group (*p*  < 0.05) and there were no significant differences between the three groups of H0, H1, and H3 (*p*  > 0.05). In H2 group, *α*-linolenic acid (C18 : 3n3), *γ*-linolenic acid (C18 : 3n6), *cis*-8,11,14-linolenic acid (C20 : 3n6), all-*cis*−5,8,11,14-eicosatetraenoic acid (C20 : 4n6), all-*cis*−5,8,11,14,17-eicosapentaenoic acid (C20 : 5n3/EPA [eicosapentaenoic acid]), all-*cis*−4,7,10,13,16-docosapentaenoic acid (C22 : 5n6), all-*cis*−4,7,10,13,16,19-docosahexaenoic acid (DHA) (C22 : 5n6/DHA) were significantly higher than those in the H0 group (*p*  < 0.05). The linoleic acid (C18 : 2n6) content was found to be higher in group H2 than that in group H0 (*p*  > 0.05). The Σ*n*−3 PUFA were found to be significantly higher in the H2 group than in the H0 group (*p*  < 0.05). The ratio of *n*−3 series to *n*−6 series PUFAs (*n*−3/*n*−6 PUFAs) was found to be higher in the H2 group than in the H0 group (*p*  > 0.05). Therefore, feeding 0.1% HT (H2) to *M. armatus* could significantly increase the content of unsaturated fatty acids in muscle, especially C18 : 1n9, C18 : 3n3, C20 : 5n3, and C22 : 5n6, and the content of *n*−3/*n*−6 PUFA was also higher than that in H0 group.

### 3.4. Digestive Enzyme Activity Analysis

The activity of digestive enzymes in the intestine and stomach of the *M. armatus* was assayed, and the results are presented in Table 7. Compared with the H0 group, the lipase activity in the stomach of the H1 group was significantly increased (*p*  < 0.05), and H2 and H3 groups were also greater than that of the H0 group, but there was no significant difference (*p*  > 0.05). The intestinal lipase activity was found to be significantly higher in all HT-added groups (H1, H2, and H3) than that in the control group (H0). The H1 and H2 groups had the highest intestinal lipase activity, and there was a statistically significant difference compared to the H0 and H3 groups (*p*  < 0.05). There was no significant difference between the H0 and H3 groups (*p*  > 0.05). There were no statistically significant differences in amylase and pepsin activities of the stomach between all groups (*p*  > 0.05). It was detected that the intestinal pepsin activity of *M. armatus* in each HT group was lower than that of the control group. However, H2 and H3 groups were significantly lower than of H0 group (*p*  < 0.05), and there was no statistical significance difference between H1 group and H0 group (*p*  > 0.05). Therefore, feeding different concentrations of HT to the diet of *M. armatus* resulted in a slight increase in lipase activity.

### 3.5. Expression Analysis of Genes

#### 3.5.1. Growth Genes in Brain

**Figure 1:**
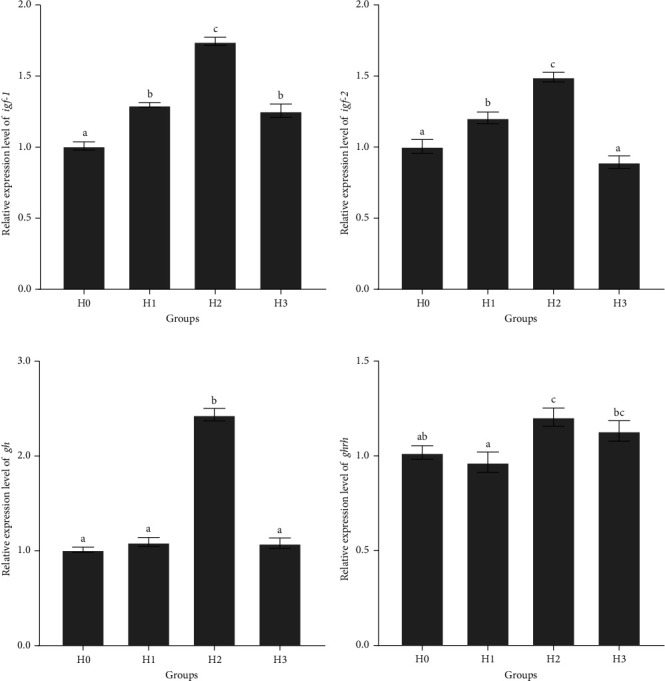
The relative expressions of *igf-1* (A), *igf-2* (B), *gh* (C), and *ghrh* (D) in the brain. *igf-1*, insulin-like growth factor 1; *igf-2*, insulin-like growth factor 2; *gh*, growth hormone; *ghrh*, growth hormone-releasing hormone. H0, basal diet; H1, basal diet + 0.05% hydrolyzable tannin; H2, basal diet + 0.1% hydrolyzable tannin; H3, basal diet + 0.2% hydrolyzable tannin. The letters in the results indicate the outcomes of multiple-range tests. The different letters indicate significant differences (*p* < 0.05), while the same letter indicates no significant differences (*p* > 0.05).

The feeding of HT significantly affected the relative expression levels of growth-related genes *igf-1*, *igf-2*, *gh*, and *ghrh* in the brain of juvenile *M. armatus* (Figure 1). The expression level of *igf-1* was significantly lower in the H0 group than that in the H1, H2, and H3 groups, and the highest expression level was detected in the H2 group (*p*  < 0.05) (Figure 1A). The expression level of *igf-2* was significantly higher in the H2 group than that in the other three groups, and the H1 group was significantly higher than that in the H0 group (*p*  < 0.05), and there was no significant difference between H3 group and H0 group (*p*  > 0.05) (Figure 1B). The expression level of *gh* in the H2 group was significantly higher than that in the other three groups (*p*  < 0.05) (Figure 1C). The expression level of *ghrh* was significantly higher in the H2 group than that in the H0 group (*p*  < 0.05), and there was no significant difference in H1 group compared with H3 and H0 groups (*p*  > 0.05) (Figure 1D). These results suggest that feeding 0.1% HT (H2) can significantly promote the growth and development of juvenile *M. armatus* by increasing the relative expression levels of *igf-1*, *igf-2*, *gh*, and *ghrh* in brain tissue.

#### 3.5.2. Hepatopancreatic Antioxidant Genes

**Figure 2:**
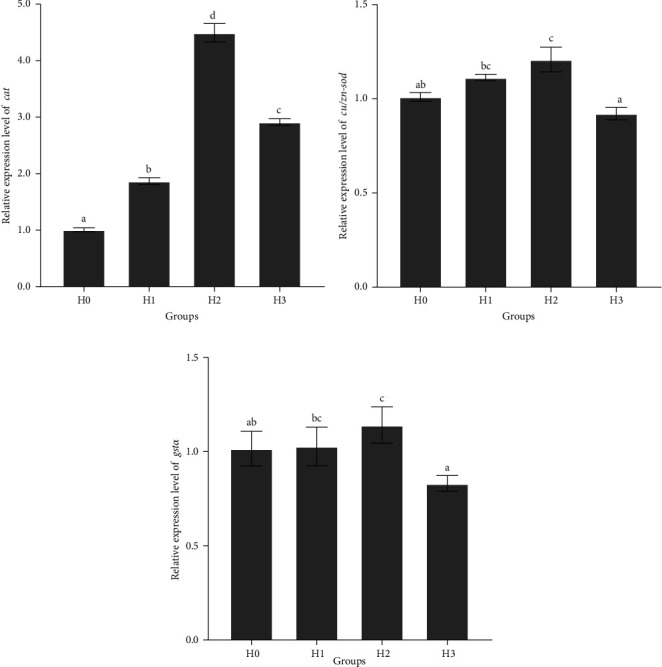
The relative expression of *cat* (A), *cu/zn-sod* (B), and *gstα* (C) in the hepatopancreas of juvenile *M. armatus*. *cat*, catalase; *cu/zn-sod*, cu/zn-superoxide dismutase; *gstα*, glutathione S-transferases *α*. H0, basal diet; H1, basal diet + 0.05% hydrolyzable tannin; H2, basal diet + 0.1% hydrolyzable tannin; H3, basal diet + 0.2% hydrolyzable tannin. The letters in the results indicate the outcomes of multiple-range tests. The different letters indicate significant differences (*p* <  0.05), while the same letter indicates no significant differences (*p* > 0.05).

The expression levels of antioxidant genes *cat*, *cu/zn-sod*, and *gstα* in hepatopancreas were detected. The results showed that the expression level of *cat* was significantly lower in group H0 than that in H1, H2, and H3 groups, and significantly higher in H2 group than that in H1 and H3 groups (*p*  < 0.05) (Figure 2A). The expression levels of *cu/zn-sod* and *gstα* were significantly higher in the H2 group than that in H0 and H3 groups (*p*  < 0.05). (Figure 2B,C). These results indicated that feeding HT to juvenile *M. armatus* enhanced the antioxidant capacity of *M. armatus* by upregulating the relative expression levels of hepatopancreas antioxidant genes *cat*, *cu/zn-sod*, and *gstα*.

#### 3.5.3. Growth Genes in Muscle

**Figure 3:**
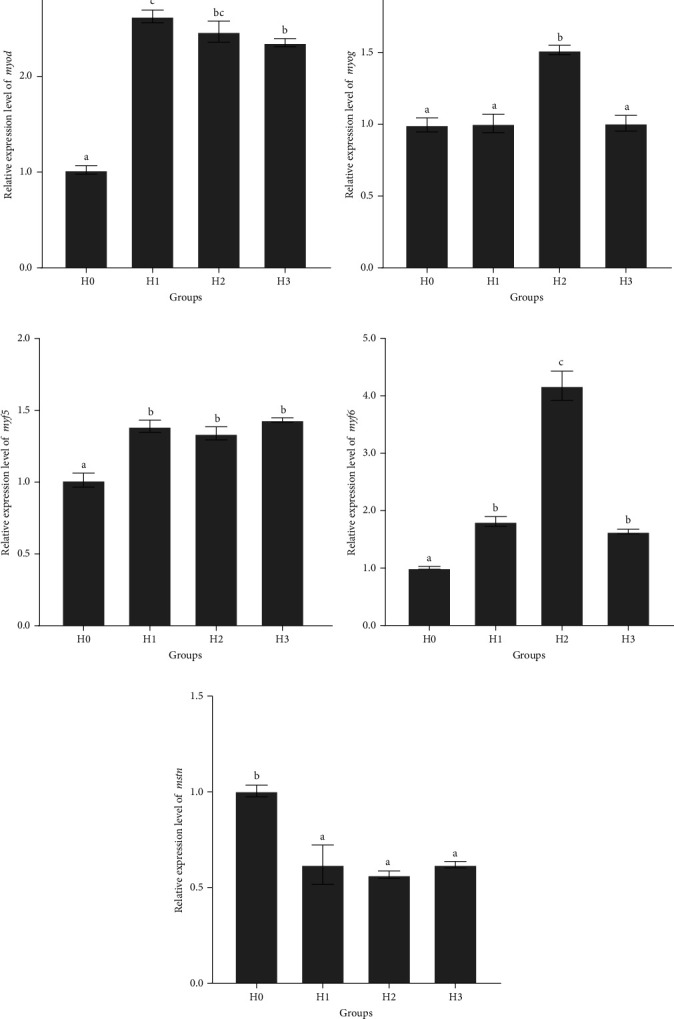
The relative expression of *myod* (A), *myog* (B), *myf5* (C), *myf6* (D), and *mstn* (E) in the muscle of juvenile *M. armatus*. *myod*, myogenic differentiation; *myog*, myogenin; *myf5*, myogenic factor 5; *myf6*, myogenic factor 6; *mstn*, myostatin. H0, basal diet; H1, basal diet + 0.05% hydrolyzable tannin; H2, basal diet + 0.1% hydrolyzable tannin; H3, basal diet + 0.2% hydrolyzable tannin. The letters in the results indicate the outcomes of multiple-range tests. The different letters indicate significant differences (*p* < 0.05), while the same letter indicates no significant differences (*p* > 0.05).

Feeding HT significantly affected the expression levels of muscle growth-related genes in juvenile *M. armatus* (Figure 3). The expression level of *myod* was significantly higher in the HT groups (H1, H2, and H3) than that in the control group (H0) (*p*  < 0.05) (Figure 3A). The expression level of *myog* was significantly higher in the H2 group than that in the other groups (*p*  < 0.05) (Figure 3B). The expression level of *myf5* was significantly higher in the HT groups than that in the control group (*p*  < 0.05) (Figure 3C). The expression level of *myf6* in the HT group was significantly higher than that in the control group. The highest level was observed in the H2 group (*p*  < 0.05) (Figure 3D). The expression level of *mstn* was significantly higher in the control group than in the HT groups (*p*  < 0.05). There was no significant difference among HT groups (*p*  > 0.05) (Figure 3E). Therefore, HT could significantly increase the expression of myogenic regulatory factor genes *myod*, *myf5*, *myog*, and *myf6*, and decrease the expression of the muscle growth inhibitor gene *mstn* in the muscle of juvenile *M. armatus*, and the effect of 0.1% HT (H2) was most significant.

## 4. Discussion

### 4.1. HT Promotes Growth of Juvenile *M. armatus*

The impact of tannins on animal growth depends on many factors, including the species of the animal, dietary structure, and specific tannin dosage administered [21–26]. In piglets [21, 22] and broilers [23, 24], the addition of HTs was effective in improving their growth performance. However, a number of studies have shown that the addition of HT to lamb [25] and broiler [26] had a negative impact on growth performance, hindered nutrient absorption and utilization, reduced feed utilization efficiency, and caused waste of nutrients. The above studies have shown that HT has a significant impact on the growth performance of livestock and poultry. Moreover, the effects of tannin are very different, even within the same species, depending on the source of the tannin, the experimental environment and the diet. In addition to its use in livestock and poultry animals, HT also has great potential application value in aquatic animals. The addition of tannin-containing *Moringa oleifera* leaf extract to the diet of *Macrobrachium rosenbergii* improved its growth performance [27]. Nevertheless, the impact of HT on the growth performance of aquatic animals is contingent upon the quantity of the additive. When excessive HT is added, there is a negative impact on the nutrient uptake and growth performance of aquatic animals [10, 28]. For instance, the incorporation of 1.5% HT into the diet of Nile tilapia resulted in a notable reduction in the rate of weight gain and specific growth rate, accompanied by a significant elevation in the feed coefficient [28]. The addition of 3% gallnut tannin to the diet of European sea bass yielded comparable results [10]. In this study, the inclusion of 0.1% HT in the diet resulted in a significant increase in survival, weight gain, and specific growth rate of juvenile *M. armatus*, and feed coefficient ratio was reduced. In conclusion, the optimal concentration of HT can promote animal growth. Although the application of HT in the growth of aquatic animals is promising, the specific mechanism of promoting growth is still large unknown. This study provides a theoretical reference for the application of HTs in the field of diet additives for *M. armatus*.

Fish growth and development is a complex biological process that is influenced by many factors that affect the neuroendocrine system. The neuroendocrine system plays a pivotal role in regulating fish growth, including the release of GHRH, GH, IGFs, and other regulatory factors [29]. In fish, both *igf-1* and *igf-2* have been demonstrated to promote myocyte proliferation and muscle growth [30, 31]. The expression level of the *igf-1* in the HT groups was significantly higher than that in the control group, and the addition of 0.1% HT to the diet was the most effective in stimulating the expression of *igf-1*. The addition of 0.05% and 0.1% HT significantly increased the expression of *igf-2* in the brain tissue of juvenile *M. armatus*, while the 0.2% HT group was slightly lower than that of the control group. These results suggest that the addition of HT in moderate amounts can help to enhance the expression of *igf-2*, but excessive addition does not. GH plays a pivotal role in fish growth and metabolism, functioning as an endocrine hormone that stimulates muscle growth, optimizes diet utilization, reduces lipid synthesis, and enhances immunity and resistance to infection [32]. In this study, the addition of 0.1% HT to the diet significantly promoted the expression of *gh*. GHRH plays a very important role in the regulation of growth and development and immune function in fish [33]. The addition of 0.1% HT to the diet in this study significantly promoted *ghrh* expression. In conclusion, feeding 0.1% HT to the diet can significantly increase the expression levels of the *igf-1*, *igf-2*, *gh*, and *ghrh* in the brain tissues of juvenile *M. armatus*, thus promoting their growth and development.

### 4.2. HT Improves Hepatopancreatic Health in Juvenile *M. armatus*

The hepatopancreas health was evaluated by detecting the expression levels of antioxidant enzymes, biochemical index-related enzyme activities, and antioxidant and immune genes in the hepatopancreas of *M. armatus*. T-SOD and CAT play a pivotal role in the scavenging of oxygen-free radicals within the homeostatic system, thus maintaining the health of organisms. They are also important antioxidant enzymes involved in the scavenging of free radicals and the reduction of oxidative stress in animals [34, 35]. T-AOC is the principal indicator for evaluating the antioxidant capacity of fish. Its magnitude reflects the capacity of the fish antioxidant system to respond to external stimuli and the homeostatic status of free radical metabolism in the body [36]. The results showed that hepatopancreatic T-SOD and CAT enzyme activities and T-AOC were significantly elevated in the *M. armatus* following the addition of 0.1% HT to its diet. Feeding HT was found to enhance the collaborative function of hepatopancreatic antioxidant enzymes in juvenile *M. armatus*, thereby ensuring the stability of oxygen radicals in *M. armatus* and providing a robust safeguard for its normal physiological processes. MDA, a byproduct of lipid peroxidation, has the potential to inflict significant damage to cell membranes. When an animal is subjected to stress and MDA accumulates in the organism, it disrupts the oxidative balance of the organism, and causes damage to the antioxidant system [37]. In the present study, the hepatopancreatic MDA content of the HT-added groups was significantly lower than that of the control group. This suggests that HT plays a beneficial role in the maintenance of hepatopancreatic health, with the greatest benefit observed when the HT content reached 0.1%. The expression levels of antioxidant-related genes can be used as an indirect indicator of the changes in antioxidant capacity of juvenile *M. armatus*. In this study, the relative expression of hepatopancreatic antioxidant genes *cat*, *cu/zn-sod*, and *gstα* were significantly increased by feeding 0.1% HT compared with the control group. These results strongly demonstrate that HT can increase the expression level of antioxidant genes, protect the health of hepatopancreas function, and thus enhance the antioxidant capacity of juvenile *M. armatus*.

ALT and AST are key enzymes in the catabolism and metabolism of amino acids in aquatic animals. They facilitate the transfer of amino acids between amino acids and ketoacids, thereby maintaining amino acid balance and stability in the body [38]. In the present study, ALT and AST in the hepatopancreas of juvenile *M. armatus* were significantly increased after feeding 0.1% HT, indicating that protein biosynthesis was not affected. In aquatic animals, AKP plays a pivotal role in the breakdown of phosphate esters, facilitating the deposition of phosphate and other substances in fish bones [39, 40]. The results of this experiment indicated that the AKP activity of the HT groups was significantly higher than that of the control group. In conjunction with growth indicators, the HT groups may necessitate a heightened level of sustained breakdown of AKP to facilitate the inorganic mineral formation required for the skeletal growth of juvenile *M. armatus*, in comparison to the H0 group.

### 4.3. HT Improves Muscle Quality in Juvenile *M. armatus*

#### 4.3.1. Muscle Amino Acid Composition

The content of flavor amino acids, such as Glu, Asp, Ala, and Gly, as well as the composition and ratio of essential and non-EAAs in aquatic animals, is important in evaluating the nutritional value of fish [41]. The addition of varying doses of HT to the diet of juvenile *M. armatus* demonstrated a discernible trend in amino acid content. Feeding 0.1% HT significantly increased Ala and Gly content. Val is a branched-chain amino acid that provides the body with energy in emergencies or harsh environments. It is also closely related to immune function, promoting the conversion of bone marrow T cells into mature T cells [42]. The addition of HT significantly enhanced the content of Val in the muscle of juvenile *M. armatus* in this experiment. This suggests that HT may play a positive role in the accumulation of Val in the muscle of juvenile *M. armatus*. His plays a crucial role in maintaining osmoregulatory processes in fish. Similar to Val, His is involved in energy production in some specific environments [43]. The addition of 0.05% and 0.1% HT to the diet of juvenile *M. armatus* was found to promote the accumulation of His in muscle. Met has been demonstrated to enhance the feed utilization of fish, exerting a regulatory effect on lipogenesis and catabolism processes. Furthermore, it has been shown to improve the antioxidant capacity through the activation of the Nrf2/Keap1 pathway, as well as enhancing the resistance to stress and pathogens through the activation of the target of rapamycin (TOR) and phosphatidylinositol 3-kinase/protein kinase B/nuclear factor-kappa B (PI3K/Akt/NF-*κ*B) pathways. These effects collectively promote the growth of fish [44, 45]. Met was found to improve the antioxidant capacity of the livers of juvenile Jian carp (*Cyprinus carpio* var. Jian) [46] and large yellow croaker (*Larimichthys crocea*) [47]. Feeding HT of juvenile *M. armatus* resulted in an increase in Met content in the muscle tissue. This finding, in conjunction with the observed growth indices and hepatopancreatic antioxidant indices, leads to the hypothesis that the addition of HT may have promoted the growth of *M. armatus*, strengthened the antioxidant defence system. In conclusion, the addition of HT will not result in a reduction of the nutritional value of muscle protein in juvenile *M. armatus*. Conversely, the addition of an appropriate quantity of HT can facilitate the accumulation of certain essential or non-EAAs and enhance the amino acid composition of the muscle of juvenile *M. armatus* to a certain extent, thereby better aligning the nutritional profile with human requirements.

#### 4.3.2. Muscle Fatty Acid Composition

The content of fatty acids in muscle is a crucial factor in evaluating the quality of meat in aquatic animals. These substances exert a significant influence on the taste of meat [48]. C18 : 1n9, a MUFA, has been demonstrated to be an effective agent in the reduction of low-density lipoprotein cholesterol levels and the prevention of atherosclerosis. Additionally, it plays an equally important role in the growth and energy supply of fish [49]. Fish fat is a rich source of long-chain unsaturated fatty acids, particularly *n*−3 PUFAs [50, 51]. These PUFAs have a beneficial impact on human health, of which C20 : 5n3 and C22 : 6n3 are involved in the regulation of the human cardiovascular system, nervous system, and immune system. They have the effect of improving brain and eyesight, enhancing memory, boosting immunity, acting as anti-inflammatories and anticancer agents, and preventing cardiovascular disease [52–54]. It is recommended that the daily diet be augmented with an increased intake of *n*−3 PUFAs, with a particular focus on C20 : 5n3 and C22 : 6n3 [55]. The incorporation of HT into the diet of grass carp resulted in an elevated PUFA content of the muscle [56]. Similar outcomes were observed when HT was incorporated into the diet of juvenile *M. armatus* at a concentration of 0.1%. The content of C18 : 1n9, C18 : 3n3, C20 : 5n3, C22 : 6n3, ΣMUFA, ΣPUFA, Σ*n*−3 PUFA, and *n*−3/*n*−6 PUFA in the muscles of the *M. armatus* was found to be higher than those of the control group that did not receive HT. This indicates that HT, as a plant extract diet additive, can effectively enhance the quality and nutritional value of *M. armatus*.

### 4.4. Effects of HT on the Expression Level of Muscle Growth-Related Genes in Juvenile *M. armatus*

The expression of *MRFs* and *mstn* is associated with muscle quality in animals. Furthermore, it has been demonstrated that these genes affect meat quality by influencing muscle proliferation and hypertrophy in fish [57]. The growth and development of myofibres are regulated by MRFs, including *myod*, *myf5*, *myog*, and *myf6* [58]. During the development of muscle cells, *myod* and *myf5* play a pivotal role in the proliferation of myofibroblast precursor cells and myofibroblasts, while *myog* and *myf6* mainly regulate myofibroblast fusion and differentiation [59]. The addition of HT to the diet of juvenile *M. armatus* resulted in the upregulated expression of *myod* in the muscle of juvenile *M. armatus*. This result demonstrates the positive regulatory effect of HT on *myod* expression in the muscle of juvenile *M. armatus*. It is postulated that HT may promote the growth and differentiation of myoblasts through the activation or enhancement of the transcriptional activity of *myod*, which in turn improves the health of the muscle tissues, and thus improves the quality of fish meat. In the experiment, feeding 0.1% HT significantly promoted *myog* expression, but the high dose of HT did not further increase the expression of *myog*. This result suggests that there was an optimal dosage range of HT to promote the growth of *M. armatus*. The regulation of *myog* and *myf6* by the upstream differentiation factors *myod* and *myf5* during skeletal muscle development [60]. Feeding 0.1% HT significantly increased the expression of *myod*, *myf5*, *myog*, and *myf6* in this study. This suggests that HT may accelerate skeletal muscle development by interacting with *myod* and *myf5*, enhancing their transcriptional activities, and thus upregulating the expression of *myog* and *myf6*. *Mstn* is a negative regulator of skeletal muscle growth [61] and acts as a negative regulator of muscle growth by inhibiting the proliferation and differentiation of fish muscle cells [62]. The expression level of *mstn* in the HT treatment groups was significantly lower than that in the control group. It is hypothesized that the HT, which is a natural substance, may have inhibited the expression of *mstn* by interacting with the *mstn* or its related regulatory elements. By reducing the expression level of *mstn*, its negative regulatory effect on muscle growth can be alleviated, thus promoting the growth and development of muscle tissue. However, the precise mechanism by which HT influences the expression of muscle growth-related factors remains unclear. Further investigation is required to elucidate the impact of the interaction between HT and muscle growth-related factors on myocyte proliferation, differentiation, and muscle growth processes. Concurrently, it is imperative to further elucidate the signaling pathways through which HT may regulate muscle growth.

### 4.5. Effects of HT on the Activity of Digestive Enzymes in Juvenile *M. armatus*

The capacity of fish to metabolize the nutrients in their diet is largely contingent upon the activity of digestive enzymes, which are frequently employed as indicators of the digestive process and nutritional status of fish [63]. Consequently, the digestive enzymes are subject to the physical and chemical properties of the diets [64]. To date, no studies have evaluated the effects of HTs on digestive enzyme activity in juvenile *M. armatus*. It is currently unclear whether the addition of HT has a damaging effect on the intestinal tract of juvenile *M. armatus*. Furthermore, the relationship between HT concentrations in diets and fish health requires further evaluation. Tannins are a highly complex class of plant secondary metabolites that are distinguished from other polyphenolic compounds by their ability to precipitate proteins. Tannins can affect protein digestion and amino acid absorption by inhibiting protease activity and forming indigestible complexes with dietary proteins [65, 66]. In the present study, the protease activity in the stomachs of juvenile *M. armatus* from the HT groups was slightly lower than that of the control group. This reduction in protease activity is presumed to be due to the combination of HT with protease, which has the effect of hindering the normal action of protease on the proteins of its substrate. Fish have a low tolerance to glucose and a limited capacity for the utilization of carbohydrates [67]. The digestion of carbohydrates in fish is largely dependent on amylases. In the present study, amylase activity was found to be the lowest of the three enzymes, protease, lipase, and amylase, indicating that carbohydrates are not effectively digested by the *M. armatus*. Lipids represent a significant source of energy for fish [68]. The increased lipase activity observed in the stomach and intestine of juvenile *M. armatus* suggests that the addition of HT results in more efficient digestion and absorption of lipid components of the diet. It is postulated that HT enhances the efficiency of nutrient absorption and utilization by increasing the activity of digestive enzymes in the *M. armatus*, thereby improving the growth performance of the *M. armatus*.

## 5. Conclusion

Feeding juvenile *M. armatus* with appropriate level of HT can significantly improve the growth performance, immune resistance, antioxidant capacity of the hepatopancreas, and protect the hepatopancreas health of juvenile *M. armatus*. The optimal supplemental level of HT in the diet is 0.1%, which could promote the growth of juvenile *M. armatus* to the maximum extent and increase the contents of EAAs and unsaturated fatty acids. HT significantly promoted the expression of myogenic regulatory factors genes and repression of the expression of myostatin gene. In addition, dietary supplementation with different concentrations of HT can increase lipase activity of *M. armatus* to varying degrees.

## Figures and Tables

**Table 1:** Composition and nutrient levels of basal diet (air-dry basis).

Ingredient (g/kg)	
Gluten flour	38.00
Brown fish meal (Chile ORIZON S.A.)	170.00
White fish meal (American Seafoods Inc.)	287.00
White fish meal (Russia)	87.00
Fermented soybean meal	120.00
Extruded soybean	25.00
*α*-Starch	248.50
Choline chloride	2.00
Calcium biphosphate	10.00
Vitamin C	1.00
Vitamin complex	2.00
Mineral matter	5.00
Taurine	1.00
Lysine	2.00
Methionine	1.50
Ingredient (g/kg)	—
Total	1000.00
Nutrient levels (%)	—
Crude protein	45.66
Crude lipid	4.00
Crude ash	17.00

*Note:* The basic diet composition and nutritional levels are provided by Dongguan Yinhua Biotechnology Co., Ltd.

**Table 2:** Primers sequences for qPCR.

Gene name	Forward primer	Reverse primer
*β-Actin*	TCATGAGGTAGTCTGTGAGGTCCC	GCCTCTGGTCGTACCACTGGTATT
*igf-1*	TGGAGATGTACTGTGCACCTGCCA	TTGTCTGGCTGCTGTGCTGTCCTA
*igf-2*	CTATTTCAGTAGGCCAACCAGC	GTGCGGGCATTACAGGAATGAC
*gh*	CTCGCTCAGAGGCTCTTCTC	TGTCTCGTGCTTGTCGATGG
*ghrh*	CGGCTGGGAGATGAAAGAGG	GCACTAGCGAAGAGGACTGG
*cat*	TGTGGCTAACTACCAGCGTG	ACTGTTGTAACGGGCCACAT
*cu/cn-sod*	ACTCCATCATTGGCCGAACC	CACCAGCATTGCCTGTCTTC
*gstα*	GCCCATATACCTGGTGGGAG	CCTCTTGATGGCGGGAATCT
*myod*	ACGCCATCAGCTACATCGAG	TCAGACCAGCGTTTGGAGTC
*myog*	GGCTACCAGGACAGGAACTC	GGCTCAGAGTGAGGTGACAG
*myf5*	AGGTCGAGATCCTACGCAAC	GAGCTGCTCTCTCCAGGTAG
*myf6*	AGAACACAGTGCCGTCAGTC	GGACTCGCTGGTTCCTTCTC
*mstn*	CAAGTGTTGAGTGTGTGGCTG	GTATCTCTCCTGGCACGCTTG

Abbreviations: *cat*, catalase; *cu/zn-sod*, cu/zn-superoxide dismutase; *gh*, growth hormone; *ghrh*, growth hormone-releasing hormone; *gstα*, glutathione S-transferases *α*; *igf-1*, insulin-like growth factor 1; *igf-2*, insulin-like growth factor 2; *mstn*, myostatin; *myf5*, myogenic factor 5; *myf6*, myogenic factor 6; *myod*, myogenic differentiation; *myog*, myogenin.

**Table 3:** Growth performance of juvenile *M. armatus*.

Group/item	H0	H1	H2	H3
Initial body weight (g)	0.40 ± 0.01	0.39 ± 0.01	0.40 ± 0.01	0.41 ± 0.01
Final body weight (g)	1.85 ± 0.06^a^	2.80 ± 0.09^b^	4.54 ± 0.13^c^	1.86 ± 0.04^a^
Weight gain rate (%)	359.35 ± 5.03^a^	632.53 ± 14.08^b^	1044.83 ± 6.33^c^	353.62 ± 8.62^a^
Specific growth rate (%/day)	2.54 ± 0.02^a^	3.32 ± 0.03^b^	4.06 ± 0.01^c^	2.52 ± 0.03^a^
Feed conversion ratio	3.12 ± 0.08^d^	1.7 ± 0.04^b^	0.9 ± 0.02^a^	2.84 ± 0.02^c^
Survival rate (%)	66.67 ± 1.75^a^	76.77 ± 2.02^b^	86.87 ± 2.02^c^	75.76 ± 1.75^b^
Condition factor (g/cm^3^)	0.27 ± 0.01^ab^	0.28 ± 0.00^b^	0.28 ± 0.01^b^	0.26 ± 0.00^a^
Visceral somatic index (%)	9.70 ± 1.26^b^	8.12 ± 0.34^ab^	6.86 ± 0.23^a^	8.53 ± 0.21^ab^

*Note:* Different superscript letters for data in the same row indicate significant differences (*p* < 0.05), while the same letters indicate insignificant differences (*p* > 0.05).

**Table 4:** Hepatopancreas parameters of juvenile *M. armatus*.

Group/item	H0	H1	H2	H3
T-SOD (U/mg prot)	98.97 ± 0.09^a^	99.99 ± 0.77^a^	122.22 ± 0.64^c^	103.50 ± 0.86^b^
CAT (U/mg prot)	5.76 ± 0.10^a^	6.58 ± 0.20^b^	7.87 ± 0.31^c^	7.14 ± 0.14^b^
MDA (nmoL/mg prot)	0.31 ± 0.01^b^	0.18 ± 0.05^a^	0.18 ± 0.01^a^	0.20 ± 0.01^a^
T-AOC (U/mg prot)	0.07 ± 0.01^a^	0.24 ± 0.01^b^	0.22 ± 0.01^b^	0.09 ± 0.01^a^
ALT (U/g prot)	46.86 ± 1.15^a^	44.26 ± 1.03^a^	58.78 ± 1.19^b^	56.94 ± 1.53^b^
AST (U/g prot)	28.12 ± 2.98^a^	31.91 ± 1.53^a^	44.62 ± 4.96^b^	31.84 ± 3.77^a^
AKP (U/g prot)	19.00 ± 2.30^a^	39.66 ± 2.11^b^	43.29 ± 1.56^b^	39.43 ± 2.68^b^
TG (mmoL/g prot)	0.02 ± 0.00^a^	0.06 ± 0.00^b^	0.09 ± 0.00^b^	0.07 ± 0.02^b^

*Note:* Different superscript letters for data in the same row indicate significant differences (*p*  < 0.05), while the same letters indicate insignificant differences (*p*  > 0.05).

Abbreviations: AKP, alkaline phosphatase; ALT, alanine aminotransferase; AST, aspartate aminotransferase; CAT, catalase; MDA, malondialdehyde; T-AOC, total antioxidant capacity; TG, triglyceride; T-SOD, total superoxide dismutase.

**Table 5:** Amino acid composition in the muscle of juvenile *M. armatus* (μg/g, wet weight basis).

Group/item	H0	H1	H2	H3
Thr	165.59 ± 13.40^c^	111.83 ± 11.33^ab^	101.95 ± 6.37^a^	137.77 ± 6.20^bc^
Val	25.78 ± 2.47^a^	36.10 ± 4.07^b^	35.81 ± 1.24^b^	39.10 ± 2.93^b^
Met	5.05 ± 0.60^a^	6.75 ± 0.77^ab^	5.96 ± 0.06^ab^	7.07 ± 0.31^b^
Phe	28.53 ± 2.45^a^	28.08 ± 1.83^a^	26.63 ± 1.73^a^	30.02 ± 3.55^a^
His	1589.25 ± 126.93^ab^	2091.95 ± 199.39^b^	1910.33 ± 218.20^b^	1257.90 ± 105.26^a^
Lys	1578.72 ± 123.86^b^	1564.99 ± 174.51^b^	1093.13 ± 73.99^a^	1962.71 ± 129.71^b^
Trp	8.88 ± 0.78^ab^	8.48 ± 0.69^ab^	8.00 ± 0.22^a^	9.96 ± 0.34^b^
Arg	131.33 ± 19.75^c^	73.44 ± 14.05^ab^	53.23 ± 0.71a	109.21 ± 19.47bc
∑EAA	3533.13 ± 193.92^ab^	3921.62 ± 130.52^b^	3235.05 ± 251.07^a^	3553.74 ± 147.79^ab^
Gly	3781.34 ± 178.39^a^	4573.42 ± 195.47^a^	4051.83 ± 204.09^a^	4176.64 ± 345.11^a^
Asp	51.77 ± 3.12^c^	15.18 ± 2.33^a^	14.80 ± 1.59^a^	30.59 ± 7.57^b^
Asn	266.33 ± 1.78^b^	230.91 ± 21.79^ab^	162.85 ± 10.59^a^	300.82 ± 47.85^b^
Ser	415.86 ± 21.08^b^	298.36 ± 31.23^a^	325.92 ± 13.62^ab^	276.58 ± 52.07^a^
Glu	141.24 ± 10.75^ab^	125.61 ± 18.58^a^	98.35 ± 3.10^a^	174.40 ± 17.98^b^
Gln	408.34 ± 24.87^ab^	396.06 ± 27.85^ab^	340.44 ± 23.61^a^	437.95 ± 28.93^b^
Ala	412.16 ± 27.31^a^	489.97 ± 41.86^a^	546.64 ± 27.43^ab^	668.38 ± 73.00^b^
Tyr	11.74 ± 0.52^a^	11.24 ± 1.59^a^	10.90 ± 0.31^a^	17.57 ± 2.40^b^
Pro	43.95 ± 3.57^b^	21.83 ± 3.31^ab^	20.50 ± 1.19^a^	27.85 ± 12.04^ab^
∑NEAA	5532.73 ± 218.33^a^	6162.60 ± 303.94^a^	5572.24 ± 222.38^a^	6110.79 ± 439.76^a^
∑AA	9065.86 ± 366.27^a^	10,084 ± 420.77^a^	8807.29 ± 447.16^a^	9664.52 ± 556.27^a^

*Note:* Different superscript letters for data in the same row indicate significant differences (*p*  < 0.05), while the same letters indicate insignificant differences (*p*  > 0.05).

Abbreviations: ∑AAs, total amino acids; ∑EAAs, total essential amino acids; ∑NEAAs, total nonessential amino acids; Ala, alanine; Arg, arginine; Asn, asparagine; Asp, aspartic acid; Gln, glutamine; Glu, glutamic acid; Gly, glycine; His, histidine; Lys, lysine; Met, methionine; Phe, phenylalanine; Pro, proline; Ser, serine; Thr, threonine; Trp, tryptophan; Tyr, tyrosine; Val, valine.

**Table 6:** Fatty acid composition in the muscle of juvenile *M. armatus* (μg/g, wet weight basis).

Group/item	H0	H1	H2	H3
C14 : 0	3.81 ± 0.24^a^	3.67 ± 0.23^a^	5.21 ± 0.43^b^	4.08 ± 0.45^ab^
C15 : 0	1.24 ± 0.04^ab^	1.10 ± 0.06^a^	1.54 ± 0.05^c^	1.28 ± 0.04^b^
C16 : 0	83.92 ± 0.65^a^	81.33 ± 2.55^a^	94.06 ± 3.00^b^	84.27 ± 3.96^a^
C17 : 0	1.66 ± 0.04^ab^	1.54 ± 0.04^a^	1.86 ± 0.01^c^	1.74 ± 0.05^bc^
C18 : 0	35.66 ± 1.47^a^	35.21 ± 0.80^a^	43.94 ± 0.31^c^	39.79 ± 1.36^b^
C20 : 0	1.44 ± 0.03^ab^	1.41 ± 0.02^ab^	1.49 ± 0.02^b^	1.41 ± 0.02^a^
C22 : 0	0.72 ± 0.01^a^	0.69 ± 0.03^a^	0.79 ± 0.01^b^	0.71 ± 0.01^a^
C24 : 0	0.78 ± 0.01^a^	0.76 ± 0.03^a^	0.78 ± 0.02^a^	0.94 ± 0.10^a^
∑SFA	129.23 ± 0.94^a^	125.71 ± 3.67^a^	149.66 ± 3.36^b^	134.22 ± 4.08^a^
C14 : 1n5	0.86 ± 0.14^a^	1.00 ± 0.12^a^	1.22 ± 0.28^a^	1.03 ± 0.12^a^
C15 : 1n5	0.51 ± 0.01^a^	0.55 ± 0.03^ab^	0.61 ± 0.06^ab^	0.69 ± 0.05^b^
C16 : 1n7	14.07 ± 1.22^a^	15.69 ± 0.43^a^	16.98 ± 1.89^a^	16.12 ± 2.04^a^
C18 : 1n12	1.13 ± 0.01^a^	1.04 ± 0.03^a^	1.03 ± 0.06^a^	1.07 ± 0.01^a^
C18 : 1n9	53.89 ± 1.39^a^	62.50 ± 4.74^ab^	69.49 ± 1.99^b^	65.60 ± 6.11^ab^
C18 : 1n7	11.46 ± 0.38^a^	11.23 ± 0.45^a^	13.20 ± 0.45^a^	12.73 ± 0.88^a^
C20 : 1n9	3.78 ± 0.04^a^	4.19 ± 0.24^a^	4.22 ± 0.16^a^	4.13 ± 0.18^a^
C24 : 1n9	3.62 ± 0.10^a^	3.47 ± 0.05^a^	3.61 ± 0.08^a^	3.63 ± 0.01^a^
∑MUFA	88.81 ± 2.56^a^	99.13 ± 5.07^ab^	109.75 ± 3.90^b^	104.30 ± 9.12^ab^
C18 : 2n6	16.93 ± 0.77^ab^	15.88 ± 1.26^a^	19.93 ± 1.06^b^	18.64 ± 0.94^ab^
C18 : 3n3/ALA	2.72 ± 0.07^a^	2.77 ± 0.18^a^	3.38 ± 0.20^b^	2.87 ± 0.14^a^
C18 : 3n6	1.24 ± 0.01^a^	1.21 ± 0.02^a^	1.33 ± 0.04^b^	1.22 ± 0.02^a^
C20 : 4n6/AA	5.43 ± 0.34^ab^	4.99 ± 0.34^a^	6.44 ± 0.05^c^	5.99 ± 0.32^bc^
C20 : 2n6	1.30 ± 0.00^a^	1.29 ± 0.09^a^	1.36 ± 0.08^a^	1.39 ± 0.06^a^
C20 : 3n3/ETE	0.93 ± 0.02^a^	0.88 ± 0.03^a^	0.92 ± 0.02^a^	0.91 ± 0.02^a^
C20 : 3n6/DGLA	0.96 ± 0.01^a^	0.91 ± 0.02^a^	1.04 ± 0.02^b^	1.04 ± 0.03^b^
C20 : 5n3/EPA	7.86 ± 0.39^a^	6.64 ± 0.59^a^	9.69 ± 0.37^b^	7.15 ± 0.33^a^
C22 : 4n6	1.42 ± 0.10^a^	1.57 ± 0.03^a^	1.67 ± 0.07^a^	1.52 ± 0.09^a^
C22 : 5n3/DPA	12.84 ± 0.65^ab^	11.02 ± 1.00^a^	14.72 ± 0.80^b^	12.94 ± 0.62^ab^
C22 : 5n6	2.22 ± 0.07^a^	2.08 ± 0.09^a^	2.53 ± 0.05^b^	2.21 ± 0.10^a^
C22 : 6n3/DHA	47.55 ± 1.56^a^	42.70 ± 2.40^a^	57.44 ± 3.22^b^	45.64 ± 0.98^a^
∑PUFA	101.40 ± 3.67^a^	91.95 ± 5.70^a^	120.46 ± 5.76^b^	101.51 ± 2.51^a^
∑*n*–3 PUFA	71.89 ± 2.61^a^	64.01 ± 3.98^a^	86.16 ± 4.48^b^	69.51 ± 1.37^a^
∑*n*–6 PUFA	29.50 ± 1.08^a^	27.94 ± 1.72^a^	34.30 ± 1.28^b^	32.00 ± 1.42^ab^
*n*−3/*n*–6 PUFA	2.44 ± 0.01^bc^	2.29 ± 0.01^ab^	2.51 ± 0.04^c^	2.18 ± 0.08^a^

*Note:* Different superscript letters for data in the same row indicate significant differences (*p* < 0.05), while the same letters indicate insignificant differences (*p* > 0.05).

Abbreviations: ΣMUFA, total monounsaturated fatty acid; Σ*n*−3 PUFA, total *n*−3 series polyunsaturated fatty acid; Σ*n*−6 PUFA, total *n−*6 series polyunsaturated fatty acid; ΣPUFA, total polyunsaturated fatty acid; ΣSFA, total saturated fatty acid; C14 : 0, myristic acid; C14 : 1n5, myristoleic acid; C15 : 0, pentadecanoic acid; C15 : 1n5, pentadecenoic acid; C16 : 0, palmitic acid; C16 : 1n7, palmitelaidic acid; C17 : 0, heptadecanoic acid; C18 : 0, stearic acid; C18 : 1n7, 11-octadecenoic acid; C18 : 1n9, oleic acid; C18 : 1n12, 6-octadecenoic acid; C18 : 2n6, linoleic acid; C18 : 3n3, linolenic acid; C18 : 3n6, gamma-linolenic acid; C20 : 0, arachidic acid; C20 : 1n9, 11-eicosenoic acid; C20 : 2n6, *cis*,*cis*-11,14-eicosadienoic acid; C20 : 3n3, all-*cis*-11,14,17-eicosatrienoic acid; C20 : 3n6, *cis*,*cis*,*cis*-8,11,14-linolenic acid; C20 : 4n6, all-*cis*-5,8,11,14-eicosatetraenoic acid; C20 : 5n3, all-*cis*-5,8,11,14,17-eicosapentaenoic acid; C22 : 0, behenic acid; C22 : 4n6, all-*cis*-7,10,13,16-docosatetraenoic acid; C22 : 5n3, all-*cis*-7,10,13,16,19-docosapentaenoic acid; C22 : 5n6, all-*cis*-4,7,10,13,16-docosapentaenoic acid; C22 : 6n3, all-*cis*-4,7,10,13,16,19-docosahexaenoic acid; C24 : 0, lignoceric acid; C24 : 1n9, 15-nervonic acid; *n*−3/*n*−6 PUFA, *n*−3/*n*−6 series polyunsaturated fatty acid.

**Table 7:** Digestive enzyme activity of juvenile *M. armatus*.

Group/item	H0	H1	H2	H3
Stomach				
Pepsin (U/mg prot)	1.06 ± 0.14^a^	0.96 ± 0.08^a^	0.85 ± 0.22^a^	0.93 ± 0.11^a^
Amylase (U/mg prot)	0.45 ± 0.03^a^	0.42 ± 0.02^a^	0.37 ± 0.08^a^	0.38 ± 0.05^a^
Lipase (U/g prot)	3.14 ± 0.75^a^	5.34 ± 0.68^b^	3.89 ± 0.25^ab^	3.32 ± 0.51^ab^
Intestine				
Pepsin (U/mg prot)	0.85 ± 0.11^c^	0.58 ± 0.04^bc^	0.15 ± 0.04^a^	0.33 ± 0.14^ab^
Amylase (U/mg prot)	0.19 ± 0.10^a^	0.14 ± 0.04^a^	0.12 ± 0.03^a^	0.17 ± 0.07^a^
Lipase (U/g prot)	3.30 ± 0.35^a^	11.49 ± 1.45^c^	8.14 ± 0.49^b^	4.03 ± 0.78^a^

*Note:* Different superscript letters for data in the same row indicate significant differences (*p* < 0.05), while the same letters indicate insignificant differences (*p* > 0.05).

## Data Availability

The data will be made available upon request.

## Nomenclature

H0: Control group
H1: 0.05% hydrolyzable tannin feeding group
H2: 0.1% hydrolyzable tannin feeding group
H3: 0.2% hydrolyzable tannin feeding group.
